# Synopses of Editorial Board

**DOI:** 10.1080/13577149878019

**Published:** 1998-06


*Sarcoma* (1998) **2**, 65–68

SYNOPSES OF EDITORIAL BOARD

### MARC LADANYI, M.D.

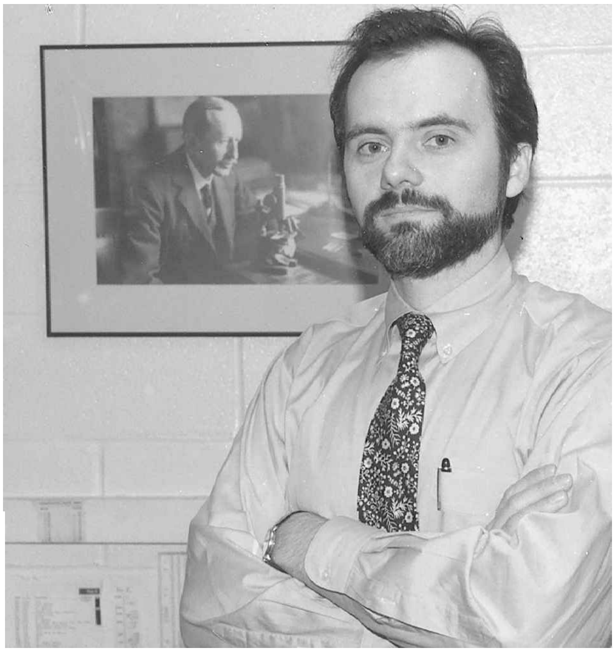

Marc Ladanyi, M.D., obtained his medical degree from McGill University (Montréal, Canada) in 1984, where he then began postgraduate training in anatomic pathology. He completed his training in tumour pathology and molecular genetics at Memorial Sloan–Kettering Cancer Center in New York City, where he is now Assistant Attending Pathologist in the Departments of Pathology and Human Genetics, and Director of the Laboratory of Diagnostic Molecular Pathology. He is a Diplomate of both the American Board of Pathology (Anatomic Pathology) and the American Board of Medical Genetics (Clinical Molecular Genetics). The focus of his current research is the basic and applied molecular pathology of sarcomas. Notable achievements of his laboratory include the co-discovery of the EWS-WT1 gene fusion in desmoplastic small round cell tumour and, more recently, the demonstration of clinical and pathological correlates of alternative fusion gene transcripts in synovial sarcoma and Ewing's sarcoma.

1357-714X/98/0200065-04 ©1998 Carfax Publishing Ltd

### OLE STEEN NIELSEN, M.D.

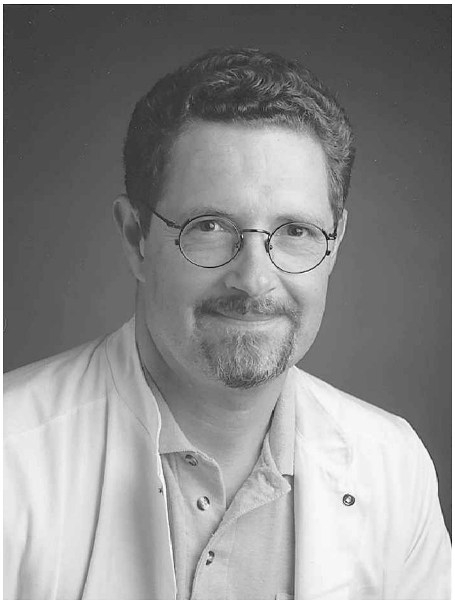

O.S. Nielsen is Head of the Department of Oncology, Aarhus University Hospital, Denmark. The department is responsible for radiation and medical oncology and medical physics, as well as experimental clinical oncology. He became a Doctor of Medical Science at Aarhus University Hospital in 1984. He has had a distinguished career in oncology, becoming Associate Professor in Oncology at the Aarhus University Hospital in 1995, where he is involved in pre- and postgraduate teaching. He has participated in the organisation of several international meetings and courses within the fields of hyperthermic oncology, radiation oncology, palliative medicine, bone metastases and sarcomas. He has been Chairman of a number of international meetings, as well as being a member of several national and international cancer societies. He was the previous Chairman of the Danish Society of Oncology, and is the present Secretary of the EORTC Soft Tissue and Bone Sarcoma Group. As well as being involved in the review of several international cancer journals, he has co-authored around 100 publications and made around 150 presentations at international meetings. Currently, his medical and clinical activities cover the treatment of anal, oesophageal and gynaecological cancers, as well as soft tissue and bone sarcomas. In addition he is pursuing research activities in the area of palliative radiotherapy and quality of life.

### DR. VIVIEN BRAMWELL

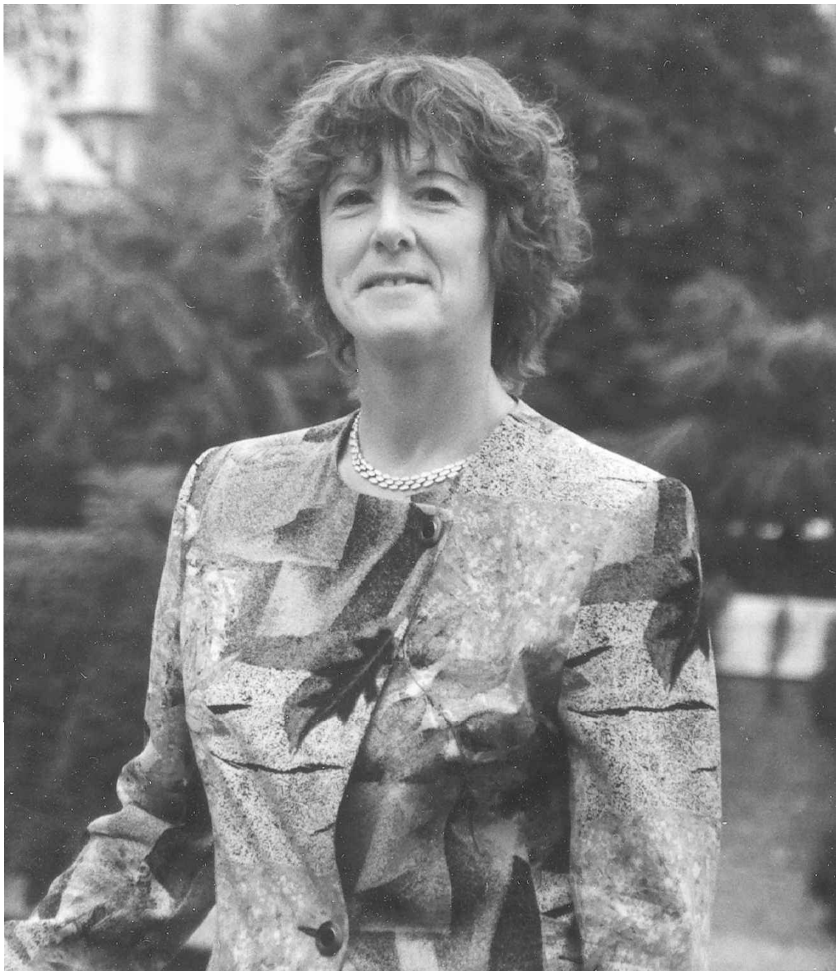

Dr. Vivien Bramwell qualified in medicine at St. Bartholomew's Hospital, London, and trained in Medical Oncology at the Christie Hospital, Manchester. She emigrated to Canada in 1984, and established the Canadian Sarcoma Group in 1985. She is currently Head of Medical Oncology at the London Regional Cancer Centre, and Professor and Head of the Division of Medical Oncology, Department of Oncology, University of Western Ontario. She has extensive experience with clinical trials in breast cancer and sarcoma, and administers the database for a Canadian soft tissue sarcoma tumor bank for molecular studies.

### ALAN CRAFT

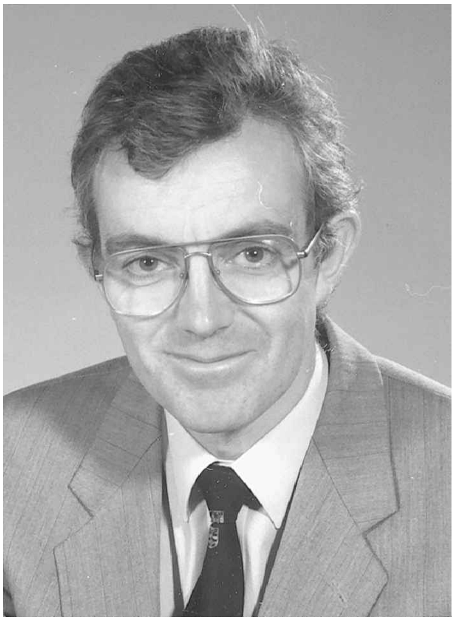

Alan Craft is currently Professor of Child Health at the University of Newcastle upon Tyne. He established the Newcastle Paediatric Oncology Unit in 1977 and was it's Director until 1992. He has a specialist clinical interest in the management of bone tumours in children and young people, and for many years was Chairman of the UKCCSG Bone Tumour Committee and the MRC Working Party on bone sarcoma. More recently he has been Chairman of UKCCSG itself. He was a founder member of the European Osteosarcoma Intergroup and is it's current Chairman. EOI has successfully completed two very large randomised trials with over 400 patients in each, the second one published in *The Lancet* in 1997 showing an equivalence between a short 2-day regime and a multidrug Rosen T10 type of regime. He is also Co-chairman of the European Intergroup Co-operative Ewing's Sarcoma study, which is close to completing its first large-scale multinational randomised trial.

